# Jefferson Medical College

**Published:** 1845-03

**Authors:** 


*Jefferson Medical College.*—American schools of medicine will soon rival, in the number of their students, those of London and Edinburgh. Four hundred and nine have been in attendance during the past season at the Jefferson College, in Philadelphia, according to the catalogue. The fact is the courtly manners of the faculty, combined with an uncommon brilliancy tact and fitness for their several stations, have given an eclat to the institution, and raised it to great and well-deserved distinction.—*Ib.*

